# Peaks of Fine Particulate Matter May Modulate the Spreading and Virulence of COVID-19

**DOI:** 10.1007/s41748-020-00184-4

**Published:** 2020-11-21

**Authors:** Mario Rohrer, Antoine Flahault, Markus Stoffel

**Affiliations:** 1 Meteodat GmbH, 8903 Birmensdorf ZH, Switzerland; 2 Climate Change Impacts and Risks in the Anthropocene, Institute for Environmental Sciences (ISE), University of Geneva, 1205 Geneva, Switzerland; 3 Institute of Global Health, Faculty of Medicine, University of Geneva, 1202 Geneva, Switzerland; 4 Swiss School of Public Health (SSPH+), 8001 Zürich, Switzerland; 5 Department F.-A. Forel for Environmental and Aquatic Sciences, University of Geneva, 1205 Geneva, Switzerland; 6 Department of Earth Sciences, University of Geneva, 1205 Geneva, Switzerland

**Keywords:** COVID-19, Thermal inversion, PM2.5 air pollution, Desert dust intrusions

## Abstract

A probe of a patient, seeking help in an emergency ward of a French hospital in late December 2019 because of Influenza like symptoms, was retrospectively tested positive to COVID-19. Despite the early appearance of the virus in Europe, the prevalence and virulence appeared to be low for several weeks, before the spread and severity of symptoms increased exponentially, yet with marked spatial and temporal differences. Here, we compare the possible linkages between peaks of fine particulate matter (PM2.5) and the sudden, explosive increase of hospitalizations and mortality rates in the Swiss Canton of Ticino, and the Greater Paris and London regions. We argue that these peaks of fine particulate matter are primarily occurring during thermal inversion of the boundary layer of the atmosphere. We also discuss the influence of Saharan dust intrusions on the COVID-19 outbreak observed in early 2020 on the Canary Islands. We deem it both reasonable and plausible that high PM2.5 concentrations—favored by air temperature inversions or Saharan dust intrusions—are not only modulating but even more so boosting severe outbreaks of COVID-19. Moreover, desert dust events—besides enhancing PM2.5 concentrations—can be a vector for fungal diseases, thereby exacerbating COVID-19 morbidity and mortality. We conclude that the overburdening of the health services and hospitals as well as the high over-mortality observed in various regions of Europe in spring 2020 may be linked to peaks of PM2.5 and likely particular weather situations that have favored the spread and enhanced the virulence of the virus. In the future, we recommended to monitor not only the prevalence of the virus, but also to consider the occurrence of weather situations that can lead to sudden, very explosive COVID-19 outbreaks.

## Introduction

In early December 2019, first cases of coronavirus disease 2019 (COVID-19), caused by the novel severe acute respiratory syndrome coronavirus 2 (SARS-CoV-2), were reported in Wuhan (China) with a “first generation” relevant human‐to‐human virus transmission probably occurring sometimes in fall 2019 (Li et al. 2020). The sudden and intense epidemiological COVID-19 storm in Wuhan, however, only started to unfold fully in January 2020. In France, a probe of a patient seeking help in hospital in December 2019 was retrospectively tested positive to COVID-19. In both countries, despite the early appearance of the virus, its prevalence and virulence thus apparently remained low for several weeks, before it increased exponentially, yet with marked spatial and temporal differences.

To inhibit spreading of COVID-19, governments have laid substantial efforts into national lockdowns, hygienic measures and social distancing. At the same time, they have largely ignored the role of viral transmission by aerosols (Smieszek et al. 2019) and/or the role of high concentrations of fine particulate matter (PM2.5) in modulating or boosting COVID-19 prevalence, morbidity and mortality. The possibility of aerosolization of SARS-CoV-2 is still vividly debated in the scientific literature (Peters et al. 2020; Dancer et al. 2020), despite ample evidence for a possible aerosolization of viruses, at least under certain conditions. Evidence also exists that the stability of SARS-CoV-2 is similar to that of SARS-CoV-1, such that both types of viruses are able to survive in aerosols for several hours (van Doremalen et al. 2020). A possible aerosol transport of several tens of meters has, for instance, been described during the SARS-CoV-1 pandemic, infecting dozens of persons, probably through airborne transport of the virus (Yu et al. 2004). Respiratory and speech droplets containing SARS-CoV-2 were found to be prone to be transported by aerosolization (Stadnytskyi et al. 2020; Morawska et al. 2020), and several cases exist for which a transmission of SARS-CoV-2 can only be explained reasonably by aerosolization (Dancer et al. 2020).

In the early 1960s, first lines of evidence started to emerge regarding an intriguing high peak of influenza morbidity and mortality and its coincidence with high concentrations of black carbon during a heavy smog situation that prevailed in London during February 1959 (Martin 1964). Therefore, the question arises whether peaks of fine particulate matter (PM2.5) concentrations can modulate or boost COVID-19 prevalence, morbidity and mortality. Peaks of PM2.5 concentrations are known to have serious deleterious health effects by their own (Díaz et al. 1999; Horne et al. 2018) and that they could thus contribute to the harmfulness of COVID-19 ‘independently’. Li et al. (2020) present first line of evidence for a link between higher aerosol optical depths and increased mortality in Germany, Italy and Spain. Similarly, Seaton et al. (1995) show that particulate air pollution (including ultra-fine particles) are able to provoke alveolar inflammation, thereby favoring the release of mediators capable of causing exacerbations of lung disease and increased blood coagulability. In a large sample of (sub-)urban patients, short-term exposure to elevated PM2.5 pollution was shown to be systematically associated with greater healthcare use for acute lower respiratory infection in children and adults (Horne 2018). Other studies (e.g., Chau and Wang 2020) argued that the concurrent occurrence of diseases from acute high levels of air pollution in various organs would indicate that the immune system attempts to connectively defend the human body from persistent and rising air pollution. In addition, evidence exists for high PM2.5 levels to even increase the relative abundance of microorganisms (Cao et al. 2014), likely also those of harmful viruses.

Noteworthy, PM2.5 and other aerosol peaks are typically linked to specific weather conditions, and more precisely to thermal air temperature inversions (Gramsch et al. 2014). It is thus possible that a given weather situation can boost the spread of a virus and have an effect on its virulence. The possible fatal role of adverse weather situations, linked aerosol peaks and related spreading of microorganisms is widely accepted in the scientific literature: The Legionella pneumophila outbreak in Portugal in 2014 (Russo et al. 2018) resulted in the infection of 377 persons of which 14 died. Here, transport of bacteria was favored by a thermal inversion situation with weak, yet persistent, unidirectional winds along with elevated levels of fine particulate matter, partially enhanced by adverse effects of a Saharan dust event (Milford et al. 2020). Dust particles transported in desert dust events have been described to contain sequences of several respiratory microbial allergens and pathogens and that the numbers of cultivatable airborne microorganisms are two to three times that found during clear atmospheric conditions (Griffin et al. 2001). The effects of combustion generated fine dust are even more harmful to human health compared to the desert dust intrusions as it may enhance the severity of influenza infection (Lee et al. 2014). The enhancement is related to the presence of elevated levels of environmentally persistent free radicals which can suppress immune cell response. In Beijing (China), research on influenza-like-illnesses (ILI) likewise suggests strong positive relationships between PM2.5 and ILI risk during the influenza season (Oct–Apr, *p*-value < 0.001), whereas no significant association was identified outside the flu season (May-Sept; Feng et al. 2016).

Thus, the question arises whether such a correlation could also exist between PM 2.5 and COVID-19 morbidity and mortality. A possible explanation for this nexus is given by Borro et al. (2020) who are attributing this relation to the PM2.5-mediated up-regulation of the SARS-CoV-2 cell receptor angiotensin-converting enzyme 2 (ACE-2). The influence of actual weather situations on the severity of COVID-19 remains debated and results have not been conclusive so far. Indications, however, clearly exist for a possible link between the unusually persistent anticyclonic situation prevailing over much of southwestern Europe in February 2020 and the favorable conditions for COVID-19 spreading in Italy and Spain (Sanchez-Lorenzo et al. 2020). Nonetheless, any attribution of COVID-19 spread and virulence to specific air temperature or humidity conditions has remained elusive so far as the virus has been shown to spread in cold or warm, humid or dry environments (Jüni et al. 2020; Yao et al. 2020).

Based on these lines of evidence and select examples presented in the following, we hypothesize that COVID-19 prevalence and virulence is linked closely to PM2.5 peaks forming predominantly during (1) the occurrence of air temperature inversions, characterized by rather cool and moist conditions, or (2) desert dust advection conditions characterized by dry and warm air masses. The aim of this paper therefore is to present several lines of evidence to corroborate the possible association between PM2.5 peaks occurring during specific weather situations and the spread and severity of COVID-19 pandemic. The hypothesis is underlined with four examples of large and serious COVID-19 outbreaks, or epidemiological storms, on Tenerife (Spain), in the Canton of Ticino (Switzerland) and in the Greater London and Paris regions and how the occurrence of elevated levels of fine particulate matter is followed by an increase in the number hospitalizations, morbidity or over mortality.

## Materials and Methods

In this paper, we compare measured time series of mean daily fine particulate matter (PM2.5) time series and data available on COVID-19 cases and/or mortality for the same regions. Analyses has been performed for Tenerife (Canary Island), London, the Swiss Canton of Ticino and the Greater Paris region. In the case of Tenerife, we have downloaded the mean daily PM2.5 values from the homepage of the regional Government of the Canaries (2020). The number of positively COVID-19 tested guests of a large hotel complex in Tenerife was available from (Hoefer et al. 2020).

In London, air pollutants remain trapped in the area during thermal inversions where they may form London’s characteristic brownish haze. Here, analysis was based on data characterizing international synoptic observations of present weather (2020) at airports inside or near London. Moreover, we downloaded daily mean PM2.5 data measured at London-Marylebone from UKAir (2020) and digitized daily COVID-19 mortality available at the homepage of the National Health Service of the United Kingdom (NHS 2020).

In the lower lying areas of the Swiss Canton of Ticino, rather shallow thermal inversions are known to form sporadically in late autumn, winter and early spring, favoring high peaks of fine particulate matter. To detect inversion situations, we consulted the international synoptic observations at the two meteorological stations Cadenazzo and Locarno-Monti from the Swiss National Weather Service MeteoSwiss (2020). Data are available from the weather service. PM2.5 data of Cadenazzo were retrieved from the Swiss National Air Pollution Monitoring Network (NABEL). COVID-19 hospitalizations and mortalities in the Swiss Canton of Ticino were provided by the Swiss Federal Department of Public Health (BAG—Cantone Ticino 2020).

For the Greater Paris area, we have taken the current weather information from publicly available synoptic data, in this case, the weather observations from the Paris-Le Bourget station (MeteoFrance 2020a), upper-air data from Trappes near Paris were provided by MeteoFrance (2020b) as well. Mean daily values of fine particulate matter (PM2.5) were provided by AirParif (2020), the mortality data by the French National Institute of Statistics and Economic Studies (INSEE 2020).

We present comparisons of the respective time-series of PM2.5 and COVID-19 morbidity and mortality and associate these with phenomena know to occur during thermal inversions such as haze or mist. The paper does not therefore present any proof for a causal link between COVID-19 morbidity and mortality and thermal inversion but hypothesizes that these synoptic meteorological situations and the related peaks in PM2.5 would be one such nexus to favor COVID-19 spread and severity.

## Results

### Massive Saharan Dust Storms and COVID-19 Outbreak in A Hotel Complex on Tenerife, Canary Island, Spain

**Fig. 1:**
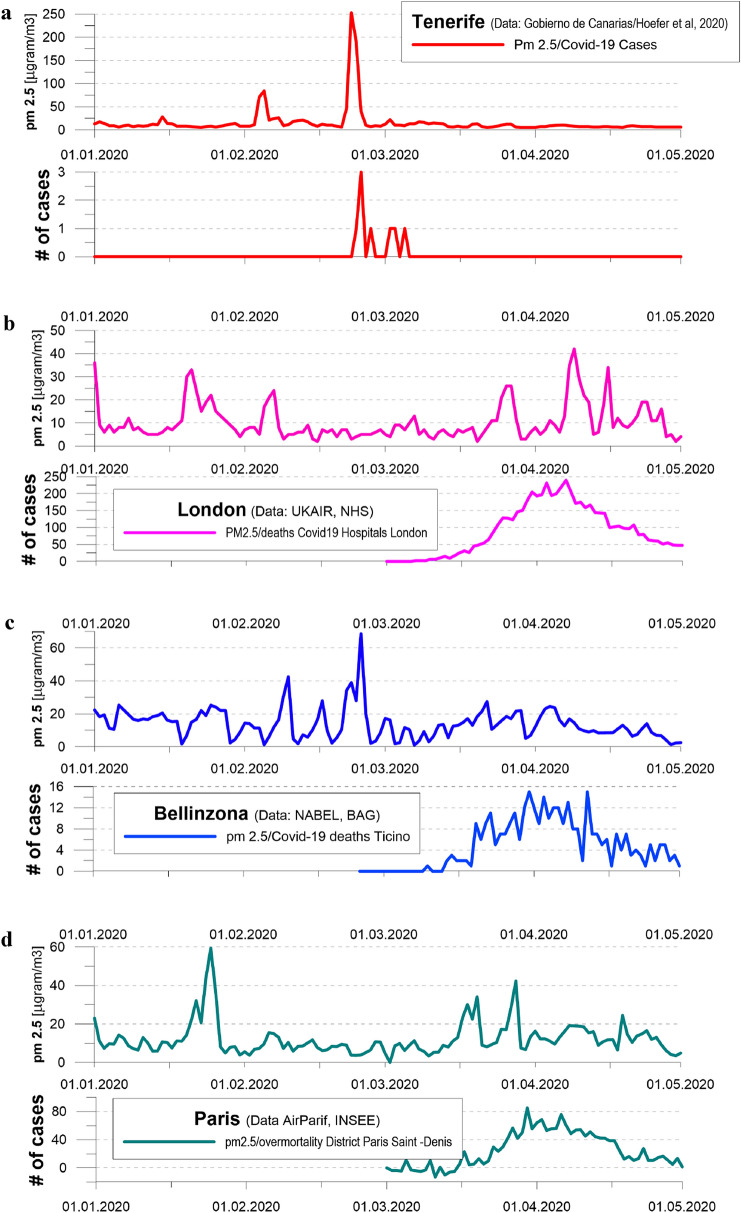
Time series of fine particulate matter concentrations (PM2.5, daily mean) and increases in reported number of COVID-19 cases for (**a**) South Tenerife where a severe Saharan dust storm occurred on February 23, 2020 and likely led to the spreading of the virus in a hotel; (**b**) Greater London area where massive haze and high PM2.5 concentrations were reported on March 26 and April 9, 2020 and the resulting COVID-19 deaths in London hospitals; (**c**) time series of PM2.5 concentrations in Bellinzona-Cadenazzo (daily mean) with a marked peak on February 24, 2020 and COVID-19 deaths in Ticino; (**d**) PM2.5 values recorded at Paris Bobigny (daily mean) and over-mortality in Paris Saint Denis

On February 23, 2020, a massive Saharan dust storm impacted the Canary Islands (Spain), leaving Tenerife blanketed in red dust and disrupting air traffic. A tourist travelling from at-risk regions in Italy fell seriously ill the day following the storm and searched help in a local hospital where he was tested positive for COVID-19. Over the subsequent days, various other hotel guests contracted COVID-19 (Hoefer et al. 2020), but the spread could be limited as the hotel (capacity > 1000 guests) was placed under quarantine with instant effect. Associations between lLI and Saharan dust storms over southern Europe are known from the literature. Furthermore, the addition of local, human-caused fine particulate matter emissions is thought to have had further negative effects on human health during dust-induced inversions (Pandolfi 2014). As these dust clouds also transport substantial amounts of *Aspergillus* fungal spores (Griffin 2007), they add to fatal interrelations between high particulate matter concentrations, *Aspergillus* spores and ILI (Yu et al. 2018). Moreover, the occurrence of aspergillosis during a COVID-19 pandemic is assumed to increase the risk to get critically ill (Verweij et al. 2020). We hypothesize that Saharan dust storms, like the one on February 23, 2020, likely have had an impact on the virulence of COVID-19 outbreaks. Figure 1a compares the time series of PM2.5 with the development of positive cases in the hotel complex under quarantine. Importantly, owing to the very quick and exemplary reaction of the hotel administration, spreading of the virus remained very limited so that only a small COVID-19 ‘wavelet’ could form.

### Haze, Fine Particulate Matter and The Flare Up of COVID-19 Mortality in The London Area

Between March and May 2020, London had the highest age-standardized mortality rate at 137.6 deaths involving COVID-19 per 100,000 persons, a value exceeding that of other regions of England in a statistically significantly manner (Iacobucci 2020). Marked increases in mortality emerge after two significant peaks of PM2.5 peaks around March 26 and April 9, 2020 that occurred during characteristic London haze situations. The haze was so dense that London airports issued warnings for incoming flights. The hurtful effect of peaks in fine particulate matter, smog and influenza-like viral infections have been known in the London area for decades (Martin 1964), and assumedly played a critical role in the spreading and virulence of COVID-19.

### A Thermal Inversion, PM2.5 Peaks and The Progression of COVID-19 Hospitalizations and Mortality in Ticino

Like in the other examples, the surge in hospitalizations and mortality in the Swiss Canton of Ticino followed a marked peak in PM2.5 concentrations. Here, the peak of fine particulate matter was supposedly enhanced by a strong thermal inversion around February 24, 2020, when morning air temperature (6 AM UTC) was 0.3 °C in the main valley (Cadenazzo), but exceeded 12 °C on the slopes (Locarno-Monti, only 160 m above the valley floor). As can be seen in Fig. 1c, the mean daily concentration of PM2.5 in Cadenzzo reached about 70 μg/m^3^ during the thermal inversion. The thermal inversion and PM2.5 peak coincided with a major carnival festivity and the gathering of 150,000 persons, which likely boosted COVID-19 prevalence and severity further. At the same time, in Zurich, north of the Alpine divide, measurements of mean daily PM2.5 concentrations did not surpass about 25 μg/m^3^ and waves of hospitalisations and mortality remained moderate.

### Mist, Thermal Inversion, PM2.5 Levels and Progression of Mortality in The Greater Paris Area

**Fig. 2:**
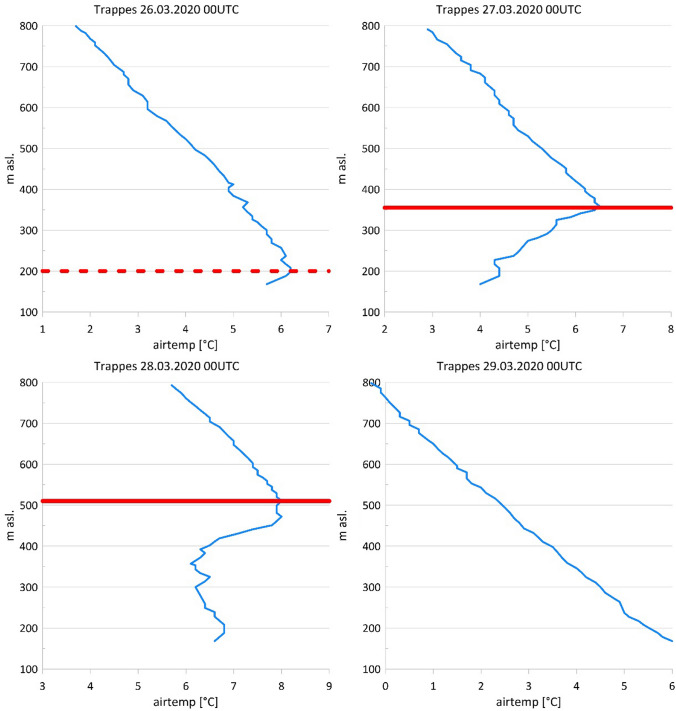
Building up and dissolution of the thermal inversion in the wider Paris area at the end of March 2020. Vertical air temperature profile as measured at 00 UTC by the radiosonde of Trappes (blue line), near Paris, from March 26 to 29, 2020 (Data: MeteoFrance, 2020b). On March 28, 2020, mean daily PM2.5 concentrations at Paris-Bobigny (Data: AirParif 2020) exceeded 40 µg/m^3^ to drop to < 10 µg/m^3^ on March 29, 2020. The height of the thermal inversion is indicated with a solid red line (and with a dashed red line for the building-up of a thermal inversion on March 26, 2020). On March 29, 2020, the thermal inversion has dissolved

Even if the first confirmed COVID-19 case was hospitalised in Paris in late 2019 (Deslandes 2020), the epidemiological storm reached the French capital only in March 2020. The storm started days after PM2.5 concentrations exceeded the threshold of 40 μg/m^3^ for the first time after mid-January 2020 in the Greater Paris area. The Paris-Le Bourget airport reported misty skies during the night and in the morning, assumedly resulting in over-mortality in one of the most affected districts (Paris–St. Denis), thereby exceeding the expected values by 2.5 times in the days following the PM2.5 peak. The corresponding evolution of the atmospheric boundary layer is depicted in Fig. 2, showing that the highest PM2.5 concentrations were recorded on March 28, 2020, i.e., on the second day of the strong thermal inversion.

## Discussion and Conclusions

The wavelet of COVID-19 in Tenerife and the epidemiological storms reported in London, Paris and the Swiss Canton of Ticino coincide or follow peaks of fine particulate matter concentrations (PM2.5). We hypothesize that the concentration of anthropogenic or desertic PM2.5 in atmospheric boundary layers has exacerbated COVID-19 morbidity and mortality in the cases presented. While we cannot rule out the possibility of serious COVID-19 outbreaks to occur in the absence of high PM2.5 concentrations, we observe interrelations among thermal inversions, PM2.5 concentrations and COVID-19 related hospitalizations and overmortality. By contrast to the assumed nexus between air temperature or humidity and COVID-19, for which evidence is generally weak or even contradictory, we suggest a dependence between the spreading and virulence of COVID-19 with the degree of air pollution (in terms of PM2.5 concentrations). We observe that during situations with a high prevalence of COVID-19 and adverse weather conditions—such as strong thermal inversions—the latter may well modulate or even boost morbidity and mortality. If thermal inversions occur in a context of high combustion-generated emissions and moderate wind speeds, PM2.5 concentrations will likely reach very high values, far beyond recommended thresholds, and thereby favor conditions that are particularly dangerous for acute lower respiratory infection (Lee et al. 2014). Based on observations and the documented nexus between high levels of fine particulate matter and the exacerbation of viral infections (e.g., Seaton et al. 1995; Jaspers et al. 2005; Robertson and Miller 2018), we conclude that suspecting high PM2.5 concentrations to aggravate COVID-19 morbidity and mortality is both reasonable and plausible.

Based on the above, we call for a more systematic forecasting and monitoring of critical weather situations—particularly thermal inversions leading to haze or fog—and high emission of combustion-generated PM2.5, as they may lead to smog and related respiratory diseases, and to vigilantly predict and track large desertic dust storms. Moreover, desert dust events likely have downwind effects on human health—even far from their sources—through the transport of fungal diseases which have been demonstrated to exacerbate the virulence and morbidity of COVID-19 (e.g., Griffin et al. 2007; Armstrong-James et al. 2020; Gangneux et al. 2020). In the absence of vaccines and to avoid future lockdowns, specific, short-term measures should be considered during these adverse weather situations so as to limit new blazes of COVID-19 morbidity and mortality. The complexity of the nexus provoking acute viral outbreaks of COVID-19 evidently encompasses physiological, social, economic, climatological and other factors, and more research across disciplines will be needed to corroborate this hypothesis further.
